# Roundtable discussion: what is the future role of the private sector in health?

**DOI:** 10.1186/1744-8603-10-55

**Published:** 2014-06-24

**Authors:** Guy Stallworthy, Kwasi Boahene, Kelechi Ohiri, Allan Pamba, Jeffrey Knezovich

**Affiliations:** 1 Bill and Melinda Gates Foundation, Seattle, WA, USA; 2 Health Insurance Fund, Amsterdam, The Netherlands; 3 Federal Government of Nigeria, Abuja, Nigeria; 4 GlaxoSmithKline, London, UK; 5 Institute of Development Studies, Brighton, UK

## Abstract

**Background:**

The role for the private sector in health remains subject to much debate, especially within the context of achieving universal health coverage.

This roundtable discussion offers diverse perspectives from a range of stakeholders – a health funder, a representative from an implementing organization, a national-level policy-maker, and an expert working in a large multi-national company – on what the future may hold for the private sector in health.

**Discussion:**

The first perspective comes from a health funder, who argues that the discussion about the future role of the private sector has been bogged down in language. He argues for a ‘both/and’ approach rather than an ‘either/or’ when it comes to talking about health service provision in low- and middle-income countries.

The second perspective is offered by an implementer of health insurance in sub-Saharan Africa. The piece examines the comparative roles of public sector actors, private sector actors and funding agencies, suggesting that they must work together to mobilize domestic resources to fund and deliver health services in the longer term.

Thirdly, a special advisor working in the federal government of Nigeria considers the situation in that country. He notes that the private sector plays a significant role in funding and delivering health services there, and that the government must engage the private sector or forever be left behind.

Finally, a representative from a multi-national pharmaceutical corporation gives an overview of global shifts that are creating opportunities for the private sector in health markets.

**Summary:**

Overall, the roundtable discussants agree that the private sector will play an important role in future health systems. But we must agree a common language, work together, and identify key issues and gaps that might be more effectively filled by the private sector.

## Background

The role for the private sector in health remains subject to much debate, especially within the context of achieving universal health coverage (UHC).

One of the reasons this debate remains so vibrant is the diversity of actors encapsulated in the term ‘private sector’. As Mills (2002) notes, the private sector in health comprises of ‘all providers who exist outside the public sector, whether their aim is philanthropic or commercial, and whose aim is to treat illness or prevent disease’ [1]. Participating in local, national and international health markets, the private sector in low- and middle-income countries ranges from informal medical practitioners and druggists to national and multi-national companies as well as non-governmental organizations (NGOs). Some of these actors are more motivated by service to the public good, while others have profit and efficiency firmly in their sights. Though these issues are not necessarily mutually exclusive, they strongly affect the types of products and services provided, the target populations for them, and their quality.

Another reason for this debate stems from the multiple and complex processes implicated in achieving UHC. While the overarching aim of UHC is to ensure that everyone has access to affordable and quality health products and services regardless of background, in practice this requires strengthening health financing for, and service provision of, an agreed package of basic health services across a broad population. Yet there remains no blueprint for how this might be achieved.

In this roundtable discussion, we present short opinion pieces from a range of relevant stakeholders offering diverse views about this complex issue. Though the comments offer suggestions on where the private sector has a complementary role to play in both health financing and on healthcare provision, we have not sought to establish an agreed view. The arguments put forward by individual commentators in this discussion are not necessarily accepted by, and may even be contradicted by, authors of other sections.

We start with a framing piece from a representative of one of the world’s largest health funders that argues that interest groups are talking past each other because they don’t share a common language. We then move on to a piece looking at the comparative roles of funders, policy-makers and private sector health funders and how they can work together. A national-level policy-maker then argues that the private sector in health is often vibrant, and that governments, therefore, must engage with these players or risk being left behind. Finally, a representative from a multinational pharmaceutical company addresses some of the opportunities and challenges for the private sector in health, focusing mainly on sub-Saharan Africa.

### Finding a common language

Guy Stallworthy, Senior Program Officer, Bill & Melinda Gates Foundation (http://www.gatesfoundation.org/).

Some people are uncomfortable using the language of markets when it comes to health. Many are acutely aware of the negative effects of market forces in health; acknowledgement of health as a right is taken to imply that the state has a duty to not only ensure access to healthcare but to provide it; financing, regulation and provision are often conflated; and public provider organizations constitute a vested interest in this respect. There is a strong preference, particularly at country level, to use the language of health systems – and subsystems – and system dynamics. On the other hand, systems thinking can be applied in ways that suggest degrees of linearity and managerial control that often do not apply. Concepts of “complex adaptive systems” correct for this.

Both kinds of language are accurate and useful. The language of markets draws attention to the fact that patients and citizens are also agents or consumers who exercise choices among sources and types of healthcare, that public and private providers are all motivated by a range of financial and non-financial incentives inherent in all provider payment systems, that laws of supply and demand apply. The benefits of both systems and market thinking can be combined when we think about healthcare as a function of complex adaptive market systems.

It is also well recognized that markets for health have particular characteristics that, while not unique to health, combine in a particular way. Information asymmetry is more acute in health transactions than in the market for, say, shoes. Barriers to entry, both regulatory and due to economies of scale, distort many health markets. To acknowledge that healthcare can be usefully understood as a market system is not to suggest that the market for healthcare is somehow “perfect”, still less that it results in socially acceptable outcomes in the absence of state intervention. On the contrary, most people acknowledge that socially acceptable health outcomes never occur without strong state action in the healthcare market, in financing, regulation and provision. A desire to use market concepts to improve equity in primary health care does not negate the need for governments to intervene in markets to ensure that they have more equitable outcomes than they do at present. It needn’t imply a desire to “promote” the private sector or to “privatize” health.

The Bill & Melinda Gates Foundation has been comfortable talking about global markets for some time in relation to specific technologies (vaccines, drugs, diagnostics, devices, vector-control products), and has been working to shape health markets for these technologies at the global level. The Foundation is also applying the concept of markets at country level – in Ethiopia, Nigeria, and in the northern states of India – to contribute to better primary health care. And it is at this level that the language begins to diverge.

However, there seems to be nothing incompatible about viewing health-care delivery as a mixed public/private market in which non-state actors are better placed to deliver some services and, at the same time, upholding the state’s ultimate responsibility for the health of its citizens.

Equally, there seems to be no inherent contradiction between seeing health as a market and, at the same time, recognizing that public finance needs to increase in both absolute terms and as a proportion of the total resources available for health. It is possible to separate finance from provision. There is consensus, for example, that collective, public or mandatory funding is the most equitable and efficient way to finance health care.

We can see the problems inherent in market-based private provision, but we also recognize the strengths of private players and markets and the dynamism presented by private markets.

Most importantly, in order to be comfortable using concepts, frameworks and analytical tools from markets we do not have to idealize market outcomes; to have respect for market forces is not to worship them but to use them as and when appropriate to achieve public policy goals.

We need to find a way to communicate about these issues, developing a language that enables us all to talk without discomfort and without alienating those who fall on one side or another. Finding ways for diverse stakeholders to come together to discuss these issues is an important next step.

### Development partners or domestic resource mobilization?

Kwasi Boahene, Director of Advocacy & Program Development at the Health Insurance Fund (http://www.hifund.org/).

Healthcare is a public good and governments have a responsibility to ensure its equitable provision. Yet in most countries in sub-Saharan Africa, those on higher incomes are more likely to have access to public-sector health services than the poorest people. Only 25% of the region’s population has access to any kind of quality health care. The reasons include: lack of investments, low public spending and limited inclusive mechanisms for pooling risks and resources. Current health expenditure per capita, for example, is standing at just $93 per year [2], compared to a developed-country (OECD) average of more than $3,400 [3].

We need a vision for effective health markets that reflects supply and demand as well as the choices made by citizens, and that minimizes the financial risks to protect both consumers and suppliers. And to achieve such a vision, governments and donors must address public spending, stimulate private investments and promote improvement in healthcare quality.

One fundamental challenge is that universal health coverage (UHC) has not been an integral part of government policies to stimulate a strong public—private partnership (PPP) for mobilizing and ensuring efficient use of resources, promoting improved delivery systems and developing local business models.

The Health Insurance Fund, a donor funded initiative working across Africa to ensure access to better quality care for low-paid and previously uninsured workers, is leveraging funding from a number of sources to address this challenge. The Fund has been building communities of practice – an area that requires investment in capacity-building, policy-making, research, and the development of local business models.

In Nigeria for example, the Fund has brought together local policy-makers, NGOs and other private sector actors to develop a health-care finance plan that will mobilize domestic resources. This has been a prime example of risk-sharing to ensure that poor people have access to good quality care while, at the same time, ensuring that health investment is worthwhile for private providers. In Lagos, the Health Insurance Fund’s subsidized health care for market women and petty traders of consumer electronics has transitioned into a non-subsidized product. In Nigeria’s Kwara state, the state government pays about 60% of the premium while the co-premium of participants has almost doubled. Private providers earn enough income from the premium to enable them to invest in quality and facilities.

Initiatives like this show that the development of health markets need not be over-reliant on development partners in the long-term. While donor funding is useful to leverage additional resources, the long-term priority must be to build strong domestic institutions that can mobilize domestic funds and, with the support of the private sector, use these funds wisely.

### Governments must intervene in health markets now or forever be playing catch up

Kelechi Ohiri, Senior Special Advisor to the Minister of State for Health in Nigeria (http://www.nigeria.gov.ng/).

Nigeria’s efforts to address health-market constraints provide pointers for policy-makers elsewhere. One key constraint has been the country’s complexity, with its devolved states running mini health-care markets and systems across 774 local government authorities and almost 10,000 wards. Health care is the responsibility of every tier of government: the risk being that if health care is everyone’s responsibility, it may become nobody’s responsibility.

The results of a recent review of Nigeria’s health markets [4] have been sobering. Drawing on consultation across the health-care space – private as well as public – the review revealed a system that was fragmented and under-performing, with sub-optimal outcomes and poor quality services that accentuated inequities. The lack of protection from financial risk undermined demand, health insurance coverage was minimal and most payments were out of pocket. The review also found that the private sector accounted for around half of all health-care provision. Clearly, it was time to stop viewing the health market as purely ‘public sector’, and recognize its mix of public and private provision. The reality, however, is that the level of engagement with the private sector was minimal, and more needed to be done to bring private players into the healthcare discourse.

As the country embarks on its aspiration toward UHC and expanding access to the poor, models of engaging the private sector – through PPPs and other mechanisms – become important for the government. In doing so, the government needs to ensure that the right governance structures are in place to ensure that equity of access to care is preserved and costs are effectively managed.

The Government aims to respond to this reality now, rather than play catch-up later. In addition to the study, the Government convened the first Private Health Summit with over 150 private health sector leaders and followed up with the establishment of a Joint Steering Task Force on Unlocking the Market Potential of the Private Health Sector. The results of these engagements suggest a role for government intervention in five key areas:

1. The review of fiscal policy (such as tariffs and import duties) that impact the health sector.

2. The improvement of regulatory frameworks through, for example, quality accreditation and standards of care.

3. Improving access to finance for healthcare investments.

4. The development of different PPP models.

5. The engagement of the private sector, beyond health, in improving access to health services.

The health sector now has a seat on Nigeria’s highest policy-making body – the Economic Management Team – and the country is developing performance-based financing mechanisms linked to disbursements. The ‘Save One Million Lives’ initiative (http://www.soml.org.ng/) uses scorecards to hold federal entities accountable for health outcomes. The Government has also set up a Ministerial Committee on Universal Health Coverage to explore options for the country as we move forward. Finally, the Government is also exploring how labor-market dynamics could attract the Nigerian diaspora to fill human-resource gaps.

The aim is to move away from ideology to focus on what works, backed by solid research and data on health markets.

### The view from ‘big business’

Allan Pamba, Director Public Engagement and Access Initiatives at GlaxoSmithKline (http://www.gsk.com/).

Given the changing business landscape, multinational companies are exploring their potential role in health markets, particularly in sub-Saharan Africa. Africa is moving up their agenda and is now seen as a region that requires robust corporate strategies. This is because Africa is now viewed as the ‘final frontier’ for business growth and companies are aware that if they are to thrive there in the coming decades, they must get in today.

There are, however, three key challenges.

First, we need a cultural shift on the ‘acceptable’ role of the private sector in health markets beyond trade or donations. We require genuine partnerships that play to the strengths of each partner – public or private – in developing and delivering solutions.

Second, we need alignment on the key gaps/priorities. Consensus will not be easy, given the number of players involved, but it is essential.

Third, healthcare is low on the agenda of many developing countries. In Africa only a handful of countries have reached the Abuja Declaration target of 15% of GDP spent on health. Without such investment, governments will find it hard to shape effective health markets.

There are, however, opportunities. The first, paradoxically, is the global economic downturn, which has created a gap in NGO health funding. This is driving greater scrutiny of existing projects, weeding out initiatives that don’t deliver and opening a potential niche for private sector participation.

Second, Africa’s growing middle class is an attractive market for the private sector. There is the growing appetite for African strategies in multinational companies that have real international ‘muscle’. The annual turnover of the top five pharmaceutical companies [5], for example, is equivalent to nearly one quarter of sub-Saharan Africa’s total GDP [6].

Third, the rising incidence of non-communicable diseases, like diabetes and hypertension, in Africa presents an interesting opportunity for the private sector; many solutions have already been developed in high-income countries and the private sector could be the bridge to carry them into low-income countries, repurposing them for local contexts.

Finally, we have new technology and innovations emerging that can help leapfrog progress and shape future health markets.

Maximizing these opportunities requires strong leadership to ensure that future markets give more people access to better health care sooner, rather than later. Next steps include the sharper framing of the health gaps to be addressed and finding good partners to plug these gaps, advocacy to raise the profile of health and greater support for health innovations that are emerging from Africa itself.

## Summary

Overall, the roundtable discussants agree that the private sector will play an important role in future health systems and in achieving UHC. However, the exact nature of the role that private sector actors might play is far from certain. Some are keen to see the role of the private sector grow, while others see working with the private sector as a pragmatic necessity in a government-dominated system. Some see the role for the private sector as focusing on service provision, while others see a distinct role for private financing. But we must agree a common language, work together, and identify key issues and gaps that might be more effectively filled by both the public and private sectors in order to achieve UHC.

## Abbreviations

UHC: Universal health coverage; NGO: Non-governmental organization; PPP: Public—private partnership; GDP: Gross domestic product.

## Competing interests

GS, KO, KW and JK declare no competing financial or personal interests. As noted in the text, AP is employed by and is a shareholder of GSK.

## Authors’ contributions

JK coordinated the production of each of the individual comments and drafted the abstract and background and summary. AP, KO, KB, and GS each prepared and developed their individual comments. Views expressed in individual sections are those of individual authors alone and do not necessarily represent the views of the other contributing authors. All authors read and approved the final manuscript.
